# Alcohol use disorder and tuberculosis treatment: A longitudinal mixed method study in Thailand

**DOI:** 10.4102/sajpsychiatry.v23i0.1074

**Published:** 2017-05-30

**Authors:** Samai Laprawat, Karl Peltzer, Wirat Pansila, Chalermpol Tansakul

**Affiliations:** 1 Faculty of Public Health, Mahasarakham University, Thailand; 2 HIV/AIDS/STIs and TB (HAST), Human Sciences Research Council, Department of Research Innovation and Development, University of Limpopo, South Africa; 3 Faculty of Public Health, Shinawatra University, Thailand

## Abstract

**Objective:**

The relationship between tuberculosis (TB) treatment and alcohol use disorders over time is under-researched. The aim of this investigation was to study alcohol use and TB medication adherence and its predictors among TB patients over a period of 6 months.

**Methods:**

A longitudinal investigation was carried out with new TB and TB retreatment patients systematically selected from two hospitals and had screened positive for hazardous or harmful alcohol use in Sisaket Province in Thailand. Alcohol use disorders were measured with Alcohol Use Disorder Identification Test (AUDIT)-C at baseline, 3 months and 6 months.

**Results:**

Of the 295 TB patients who were screened with AUDIT-C, 72 (24.4%) tested positive for hazardous or harmful alcohol use. At 6 months, 72 TB patients had completed the follow-up. At the 6-month follow-up, hazardous or harmful drinking was reduced by 84.7%. Multivariate logistic regression analysis using generalised estimation equation modelling found that alcohol use significantly reduced over time, whereas there was no change in current tobacco use.

**Conclusion:**

The prevalence of alcohol use disorders significantly reduced over a period of 6 months.

## Introduction

Thailand is a high-burden tuberculosis (TB) country; the TB case detection rate was 80.0% and the TB treatment success rate was 81.0%.^1^ In Thailand, the age-standardised prevalence of hazardous or harmful alcohol use was 16.6% among men and 2.1% among women, and current smokers were 45.8% among men and 2.3% among women.^2^ In a study among male hospital outpatients in central Thailand, the proportion of moderate or high-risk alcohol users was 44.2%, and the proportion of moderate or high-risk tobacco users was 55.8%.^3^

In cross-sectional studies, high prevalences of alcohol use disorders have been found among TB patients.^4,5^ Alcohol use has been found associated with TB treatment default,^6,7,8,9^ multidrug-resistant TB^10^ and the hazard for an unsuccessful outcome of multidrug-resistant TB.^11,12^ It is unclear whether alcohol disorders reduce, stay the same or increase after initiating TB treatment. There is a lack of longitudinal studies on alcohol use disorders in TB patients during their TB treatment, and the nature of comorbidity between alcohol use and other mental disorders such as depression and smoking during TB treatment is not well understood.^13^ For example, in a brief alcohol intervention trial among TB patients in South Africa, alcohol use reduced significantly from the beginning of TB treatment to 6-month follow-up in the control group.^14^

In relation to other mental disorders, including major depressive disorder, which often co-exist with alcohol use disorders, several studies have found an improvement of mental health scores during the course of TB treatment.^15,16,17,18,19,20,21,22^ Some of the researchers^19,20,21,22^ explain this decline in mental symptoms with the general physical improvement during TB treatment that subsequently improves mental well-being. Regarding tobacco use disorders, another comorbidity of alcohol use disorders, two smoking intervention studies in Nepal and Pakistan found no reduction of smoking in the control group.^23,24^ The results from these studies seem to support the idea that routine interventions by health care providers do not prevent tobacco use among TB patients. However, in a large alcohol intervention trial with TB patients in South Africa, daily or almost daily tobacco use significantly reduced over time during TB treatment.^25^

The relationship between TB and alcohol use during the time of TB treatment needs more research. Therefore, the aim of this investigation was to estimate the prevalence of alcohol use disorder severity and its predictors among TB patients over a period of 6 months in Thailand.

## Methods

### Study design

This is a longitudinal, mixed observational study of TB patients with hazardous or harmful drinking problems in two public district hospitals in Sisaket Province in northeastern Thailand.

### Sample and procedure

Adult new TB and new retreatment patients with hazardous or harmful alcohol use from two hospitals in Sisaket Province in Thailand were consecutively interviewed after informed consent was obtained within 1 month of starting anti-TB treatment and were followed up at 6 months of treatment in 2015–2016. There were no exclusion criteria. Quantitative information was collected for 72 patients, and qualitative information was collected for a sub-sample of 20 patients. Every third TB patient recruited for the quantitative interview was included for the qualitative interview until a sample size of 20 was reached. Quantitative and qualitative individual interviews were conducted in Thai language. The measures for alcohol use and depression were available in Thai language, and the remaining sections were translated from English to Thai and back-translated by two independent bilingual translators.

## Measures

### Quantitative

Hazardous or harmful alcohol use was assessed with the Alcohol Use Disorder Identification Test (AUDIT)-C, using a cut-off score of 3 for women and 4 for men.^26^ The Thai version of the AUDIT has been validated in Thailand.^27^ The Cronbach alpha for the AUDIT-C in this study was 0.89 at Time 1, 0.97 at Time 2 and 0.92 at Time 3.

Tobacco use was assessed with two questions: (1) ‘Do you currently smoke any tobacco products, such as cigarettes, cigars or pipes?’ and (2) ‘Do you currently use any smokeless tobacco, such as snuff, chewing tobacco, betel?’ Response options were ‘yes’ or ‘no’.^28^

Depression was assessed with a Thai-validated nine-item Primary Health Questionnaire (PHQ-9) (Thai version – local language).^29^ A score of 10 and more was used to classify moderate-to-severe depressive symptoms. The Cronbach alpha for the PHQ-9 in this study was 0.70 at Time 1, 0.82 at Time 2 and 0.72 at Time 3.

Anti-TB medication adherence was assessed with the question, ‘In the past 10 days, on how many days did you not take your TB medication?’ Taking less than 90% anti-TB medication was classified as non-adherence to TB medication.^30^

Health variables such as TB treatment status and HIV status were assessed by interviews and from medical file information.

Socioeconomic characteristics included age, gender, educational level and monthly family income.

TB treatment outcomes were defined as successful treatment (defined as cured or completed treatment) or unsuccessful treatment.^31^ The TB treatment protocol in Thailand follows WHO guidelines, including directly observed treatment (DOT) support, and provides health education on substance use.^31^

### Qualitative

Guiding questions were adopted from Thomas et al.^32^ as follows:

In your opinion does alcohol use affect TB treatment compliance? What do you perceive as the problems you encounter because of alcohol use? Could you elaborate on reasons why you might have missed your TB medication because of alcohol use? (p. 3)

### Data analysis

Data were analysed using the IBM Statistical Package for the Social Sciences (SPSS) for Windows version 23.0 (SPSS, Inc., Chicago, IL, USA). Frequencies, means and standard deviations were computed to describe the sample. Chi-square and Friedman tests were performed to compare repeated measures of alcohol use and depressive disorders at baseline and at 6-month follow-up. Multivariate logistic regression analysis was conducted using generalised estimation equation (GEE) modelling. Probability < 0.05 was regarded as statistically significant. Qualitative data were analysed using thematic content analysis.

## Results

### Sample characteristics

Of the 295 TB patients who were screened with AUDIT-C, 72 (24.4%) were found to be positive for hazardous or harmful alcohol use. At baseline, 72 TB patients with hazardous or harmful drinking and, at 6-month follow-up, 72 TB patients were included. Most of the participants were men (90.3%), 9.7% were women, 75.0% were 41 years and older, 77.8% were married, 84.7% had a primary or less education and 34.7% had a monthly family income of 8000 or more Thai Baht. Regarding health variables, 97.2% of the sample were new TB patients, 2.8% were retreatment TB patients and 6.9% were HIV positive (Table 1).

**TABLE 1:** Sample characteristics at 6 months of follow-up (*N* = 72).

Socio-demographics	*N*	%
**Gender**		
Male	65	90.3
Female	7	9.7
**Age**		
18–40	18	25.0
41–54	30	41.7
55 or more	24	33.3
**Marital status**		
Married	56	77.8
Single, widowed, separated, divorced	16	22.2
**Education**		
Primary or less	61	84.7
Post primary	11	15.3
**Family income (monthly in Thai Baht)†**		
0–4999	18	25.0
5000–7999	29	40.3
8000 or more	25	34.7
**Health variables**		
New TB patient	70	97.2
Retreatment TB patient	2	2.8
HIV negative	67	93.1
HIV positive/coinfected	5	6.9

TB, tuberculosis.

† One US$ = 36 Thai Baht.

### Alcohol use, tobacco use, depression and tuberculosis medication non-adherence at baseline, 3-month and 6-month follow-up

At baseline, the mean AUDIT-C score was 8.6, which significantly reduced to 1.3 at 3 months and 1.0 at 6 months. The proportion of hazardous or harmful alcohol users also reduced significantly over time from 100.0% to 15.3%. Similarly, past month binge drinking reduced from 88.9% at baseline to 13.9% at 6-month follow-up. Almost one in five (18.1%) were current tobacco users at baseline, which remained almost the same over time (24.1% at 3 months and 15.5% at 6 months). Moderate or severe depression significantly reduced from 25.0% at baseline to 11.1% at 6-month follow-up. TB medication non-adherence was 4.2% at baseline and 6-month follow-up. Of the 72 TB patients, 70 successfully completed the treatment and 2 defaulted (Table 2).

**TABLE 2:** Change in alcohol, tobacco use, depression and TB medication non-adherence over 6-month follow-up (N = 72).

Variables	At baseline (*N* = 72)		3 months (*N* = 30)		6 months (*N* = 72)		Change from baseline to 6 months (*M*/%)	*p*
	*M* (SD)	*N* (%)	*M* (SD)	*N* (%)	*M* (SD)	*N* (%)		
AUDIT-C score	8.6 (2.5)		1.3 (3.6)		1.0 (2.6)		−7.6	<0.001
Hazardous or harmful alcohol use		72 (100)		6 (20.0)		11 (15.3)	−84.7	<0.001
Binge drinking (past month)		64 (88.9)		6 (20.0)		10 (13.9)	−75.0	<0.001
Current tobacco use		13 (18.1)		7 (24.1)		11 (15.5)	−2.6	ns
Moderate or severe depression (PHQ-9 = ≥10)		18 (25.0)		11 (36.7)		8 (11.1)	−13.9	<0.001
TB medication non-adherence		3 (4.2)		2 (6.7)		3 (4.2)	0.0	ns
Treatment outcome	70 completed or cured 2 defaulted							

AUDIT-C, Alcohol Use Disorder Identification Test; PHQ-9, nine-item Primary Health Questionnaire; TB, tuberculosis.

### Predictors of hazardous or harmful drinking and binge drinking

In multivariate logistic regression analysis using GEE modelling, hazardous or harmful drinking and binge drinking significantly reduced over time. Further, current tobacco was positively associated with hazardous or harmful alcohol use and binge drinking. Due to too few cases of women, TB non-medication adherence, HIV status and TB treatment outcome (default vs. completed or cured); these variables were not included in the model (Table 3).

**TABLE 3:** Predictors of hazardous or harmful drinking and binge drinking.

Variables	Hazardous or harmful drinking	Binge drinking
	AOR (95% CI)†	AOR (95% CI)‡
**Socio-demographics**		
Age in years		
18–40	1 (Reference)	1 (Reference)
41–54	0.93 (0.86–1.01)	0.93 (0.81–1.06)
55 or more	1.00 (0.93–1.08)	1.05 (0.91–1.21)
**Marital status**		
Single, widowed, separated, divorced	1 (Reference)	1 (Reference)
Married	1.03 (0.93–1.13)	1.02 (0.87–1.18)
**Education**		
Primary or less	1 (Reference)	1 (Reference)
Post primary	0.96 (0.86–1.07)	0.93 (0.78–1.09)
**Current tobacco use**		
No	1 (Reference)	1 (Reference)
Yes	1.16 (1.02–1.33)*	1.24 (1.09–1.42)***
Depression (moderate to severe)	1.16 (1.02–1.33)	0.97 (0.81–1.15)
**Time**		
Baseline (≤ 1 month on TB treatment)	1 (Reference)	1 (Reference)
Time 2 (6 months)	0.43 (0.40–0.47)***	0.47 (0.43–0.53)***

*** *p* < 0.001; *p < 0.05

AOR, adjusted odds ratio; CI, confidence interval.

† Goodness of fit: quasi-likelihood under independence model criterion (QIC): 23.21.

‡ Goodness of fit: QIC: 31.18.

### Qualitative results

The qualitative results can be classified into three groups: (1) drinkers who reduced drinking at the beginning of TB treatment but increased after improving, (2) drinkers who eventually stopped drinking and (3) drinkers who continued drinking:

(1) In a number of patients, there was a tendency to reduce or even stop drinking alcohol at the beginning of TB treatment, , but after 3 or 4 months of treatment, when their general health improved, they resorted to increased use of alcohol:

‘Sometimes I accidentally drank a lot and forgot taking the pills.’ (Man, 39 years old)

‘After work I used to drink with colleagues, but not so frequently. When I felt sick, I used I quit drinking. After my body [*physical well-being*] had improved, I started drinking with my friends again.’ (Man, 35 years old)

(2) Another group of patients stopped drinking and their relationship with the family and community improved:

‘I stopped drinking since I got TB. The doctor recommended not drinking alcohol in order to recover. I am aware that alcohol can cause side effects of the drugs harming my body.’ (Man, 49 years old)

‘I used to drink before going to the clinic. The health officers smelled alcohol and complained that I had to stop drinking. I explained that I would try, but then later I stopped drinking.’ (Man, 38 years old)

(3) A third group of patients continued drinking alcohol:

‘I continued drinking until the next morning. This gives me trouble and affects my drug taking.’

‘Alcohol affects you. I do not think about it. I have a drinking problem; I live apart from my wife. Some days I forget to take my medicine.’

## Ethical consideration

The study protocol was approved by the Faculty of Public Health, Mahasarakham University Ethics Committee (No. PH 003/2559) and the Ministry of Public Health of Sisaket Province, Thailand.

## Discussion

This prospective cohort study found a significant decline in the prevalence of alcohol use disorder among TB patients over a 6-month period, as also found in the control group of a brief alcohol intervention trial among TB patients in South Africa.^14^ Possible explanations for the decline of alcohol use in this study may be attributed to (1) the intervention effect of standard care (health care worker’s advice to reduce or stop drinking alcohol), (2) the negative experience of drinking and taking anti-TB medication, as found from the qualitative interviews in a sub-sample of the studied TB patients and (3) natural history changes in drinking over time in the course of TB treatment.^33^

Further, this study in agreement with several previous studies^15,16,17,18,19,20,21,22^ found that mental well-being improved or depressive symptoms reduced during the course of TB treatment.

The reduction of depressive symptoms could be explained by the improvement of the physical health status over the duration of treatment, which then could also improve the mental well-being.^19,20,21,22^

However, this study found that tobacco use remained unchanged over the course of TB treatment. This finding is in agreement with two previous studies^23,24^ and in disagreement with another study that found a decline of tobacco use during TB treatment,^25^ while, in a study in Indonesia, most TB patients were found to stop smoking when under TB treatment but a large group would relapse to smoking post-treatment.^34^ In multivariable analysis, tobacco use but not depression symptoms was associated with hazardous or harmful alcohol use and binge drinking in this study. A high degree of association between alcohol and nicotine dependence has been pointed out earlier.^35,36^ As both alcohol and tobacco use, in particular, negatively impact on TB treatment, dual interventions including alcohol and tobacco may be indicated.^36^ Other authors^37^ suggest to better integrate TB and mental health services.

### Study limitations

Generalisability of our findings is limited to TB patients with alcohol problems on anti-TB treatment in hospital facilities in Sisaket Province in Thailand. A further limitation was that most study variables were assessed by interviews, and socially desirable responses may have been provided.

## Conclusion

The prevalence of alcohol use in this study among TB patients significantly reduced over a 6-month period.
